# Twenty-Four-Hour and Annual Variation in Severity of Pyrexia: A Retrospective Observational Study of Children Attending a Community Pediatric Emergency Department

**DOI:** 10.7759/cureus.109156

**Published:** 2026-05-18

**Authors:** Ami P Shah, Michael H Smolensky, Shahab Haghayegh, My Tam N Phan

**Affiliations:** 1 Department of Emergency Medicine, University Medical Center of Southern Nevada, Las Vegas, USA; 2 Department of Biomedical Engineering, Cockrell School of Engineering, The University of Texas at Austin, Austin, USA; 3 Department of Cardiology, McGovern Medical School - UTHealth Houston, Houston, USA; 4 Department of Anesthesia, Critical Care and Pain Medicine, Massachusetts General Hospital, Boston, USA; 5 Division of Sleep Medicine, Harvard Medical School, Boston, USA; 6 Department of Sleep Medicine, Broad Institute of Massachusetts Institute of Technology (MIT) and Harvard, Cambridge, USA; 7 Department of Emergency Medicine, Kirk Kerkorian School of Medicine, University of Nevada, Las Vegas (UNLV), Las Vegas, USA

**Keywords:** annual rhythm, body temperature, children, circadian rhythm, fever, pediatric emergency department, pyrexia

## Abstract

Background

The body temperature (BT) circadian rhythm (BTCR) of healthy, full-term neonates becomes clearly discernible by six months of age and is prominent by two to five years of age, with the evening peak-to-morning trough 24-hour variation of ~1°C.

Methods

We retrospectively reviewed the triage BT data of children aged six months to six years presenting with pyrexia - temporal or oral temperature ≥100.4°F/38°C - to a community pediatric emergency department (PED) between January 1, 2018, and June 30, 2023, to explore evidence of the persistence of the BTCR during fever.

Results

A prominent sinusoidal-like 24-hour pattern in the hourly mean and median triage BT measurements of 5,953 children was substantiated by Cosinor analyses (always p < 0.0001). Severity of pyrexia, in terms of the hourly mean and median BT values, was greater by ~0.28°F (~0.16°C) and ~0.42°F (~0.23°C), respectively, in children presenting to the PED between 21:00 h and 02:00 h than in those presenting between 07:00 h and 11:00 h. Mean and median BT values also exhibited annual patterning, with the highest values observed in November through January, and the lowest ones in June through August.

Conclusion

These findings support the hypothesis that the endogenous BTCR persists during fever, with pyrexia more severe in the late evening than in the morning, consistent with the findings from prior studies of hospitalized patients. The detected annual variation in the severity of pyrexia suggests the impact of exogenous phenomena, particularly seasonal differences in the occurrence and severity of infectious diseases.

## Introduction

Along with blood pressure, heart rate, and respiratory rate, body temperature (BT) serves as a vital sign of significant clinical utility. The BT of healthy individuals is not constant but varies as predictable-in-time 24-hour [1-5], annual [6-9], and menstrual cycle patterns [10-13]. The BT circadian (~24-hour) rhythm (BTCR) evolves over time following birth, becoming increasingly distinct during infancy and early childhood [14-18]. By two years of age, the defining features of the BTCR - 24-hour mean, amplitude, and phasing - resemble those of adolescents and young to middle-aged adults. Its pattern is characterized by a decline in the hour preceding the customary time of sleep onset, an absolute minimum toward the end of the sleep span, a gradual rise just before sleep offset (awakening), a further upswing during the initial hours of wakefulness, a plateau during the daytime with a slight midday dip, and an absolute peak approximately four to six hours before usual bedtime [15].

Pyrexia is one of the most common medical conditions of young children encountered by pediatricians. Although its severity does not necessarily reflect the clinical severity of illness, because the BT of febrile children commonly fluctuates during the 24 hours and sometimes exhibits alarmingly high levels, caregivers often become anxious. Whether this time-of-day variability in the severity of pyrexia in infants and young children reflects the persistence of the BTCR during fever remains unclear. Except for the investigation involving children by Lell et al. [19], prior studies of potential relevance focused more on time-of-day assessment strategies to discern the onset and intensity of pyrexia in hospitalized adults rather than on the persistence of the BTCR in the febrile state [20-32].

Understanding the circadian behavior of BT in fever is directly relevant for pediatricians and emergency medical providers and is important for educating and counseling parents and caregivers. Additionally, recognition of the 24-hour pattern of BT in the febrile state enables effective resource allocation of the pediatric emergency department (PED) to optimize staffing, improve triage strategies, and enhance patient care. Given the limited literature concerning time-of-day and time-of-year differences in the severity of pyrexia in young children, the primary objective of this study was to examine circadian (24-hour) variation in fever presentations among PED patients. We performed a search of the medical records of our PED for the clock time and calendar date of fever presentations - defined as a triage temporal or oral temperature ≥100.4°F/38°C in patients aged six months to six years. We focused on this age range because of our concurrent research on childhood febrile epilepsy, which occurs in children of this age [33,34]. Secondary objectives included evaluating annual (seasonal) variation in fever severity and considering potential clinical implications of observed temporal patterns.

## Materials and methods

This study was a retrospective observational analysis conducted at a university-affiliated children’s hospital. Approval was obtained from the Institutional Review Board of our university children’s hospital to search, using the EPIC software system, the electronic medical records of 131,870 children who attended the PED between January 1, 2018, and June 30, 2023.

Inclusion criteria were medical records of girls and boys aged six months to six years diagnosed with fever - defined as a triage temporal or oral temperature ≥100.4°F (38°C) - and complete data for all specified study variables. Exclusion criteria were children presenting to the PED without fever, age younger than six months or older than six years, and an incomplete medical record.

A structured, retrospective electronic medical record review was conducted. Extracted information included triage BT (°F), date of birth, clock time of BT measurement, calendar date of the PED encounter, and medical record number. BT was measured primarily by the EXERGEN temporal artery scanner system; it was measured infrequently with the Welch-Allyn oral digital system only when temporal artery measurement was not feasible or BT required verification. While the use of two measurement modalities may introduce measurement variability, both methods are widely accepted in pediatric emergency care and represent real-world practice. Information regarding prior antipyretic use was not reliably available in this retrospective dataset and, as such, was not included in the analysis.

BT data were sorted by clock hour and by month to derive mean and median hourly and monthly values. The resulting 24-hour and 12-month time series, which were closely sinusoidal in form, were analyzed by Cosinor analysis in R Studio (version 2024.12.1+563; RStudio, Inc., Boston, MA, USA) to determine the statistical significance of the respective temporal patterns and to describe them in terms of the single cosine model-estimated Midline Estimating Statistic of Rhythm (MESOR, the 24-hour or annual time series mean BT around which the temporal variation occurs), acrophase (the clock time or month of the peak BT), and amplitude (the total peak-to-trough difference in BT during the 24 hours or year).

Cosinor analysis involved approximation of a 24-hour and 365.25-day cosine curve to the respective clock-time and monthly mean and median BT time series data by the method of least squares to test the null hypothesis that the amplitude of temporal variation during the corresponding time periods is zero, i.e., the absence of sinusoidal-like oscillation [35,36]. This null hypothesis was assessed by an F-test of the amount of variance accounted for by approximation of the single cosine curve of period equal to 24 hours or 365.25 days versus that accounted for by the approximation of a straight line to the respective time series data. Rejection of the null hypothesis at p < 0.05 was considered evidence of statistically significant sinusoidal-like temporal patterning.

Interpretation of the findings of Cosinor analyses, particularly regarding the circadian acrophase and amplitude values, requires identification of the 24-hour temporal pattern of sleep and wakefulness of the sample, since it acts as a major synchronizer of circadian rhythms. Although the clock time of sleep and wakefulness of the individual patients was not recorded, we assumed the children adhered to age-specific recommended schedules.

Nighttime sleep is expected to commence in six-month-olds to one-year-olds between 18:30 and 19:30 h, in two- to three-year-olds around 19:00-20:00 h, and in six-year-olds between 19:00 and 21:00 h, with daytime wakefulness commencing across age groups between 06:00 and 07:00 h. Diurnal napping duration varies by age, with two-year-olds typically napping up to three hours and four-year-olds napping approximately 1-1.5 hours. 

## Results

Complete medical records of 5,953 children aged six months to six years were available for all study variables. The most common causes of pyrexia were influenza (N = 2,001), respiratory infections (N = 1,588), ear, nose, and throat infections (N = 696), and gastrointestinal infections (N = 452).

Circadian (24-hour) variation in BT

**Figure 1:**
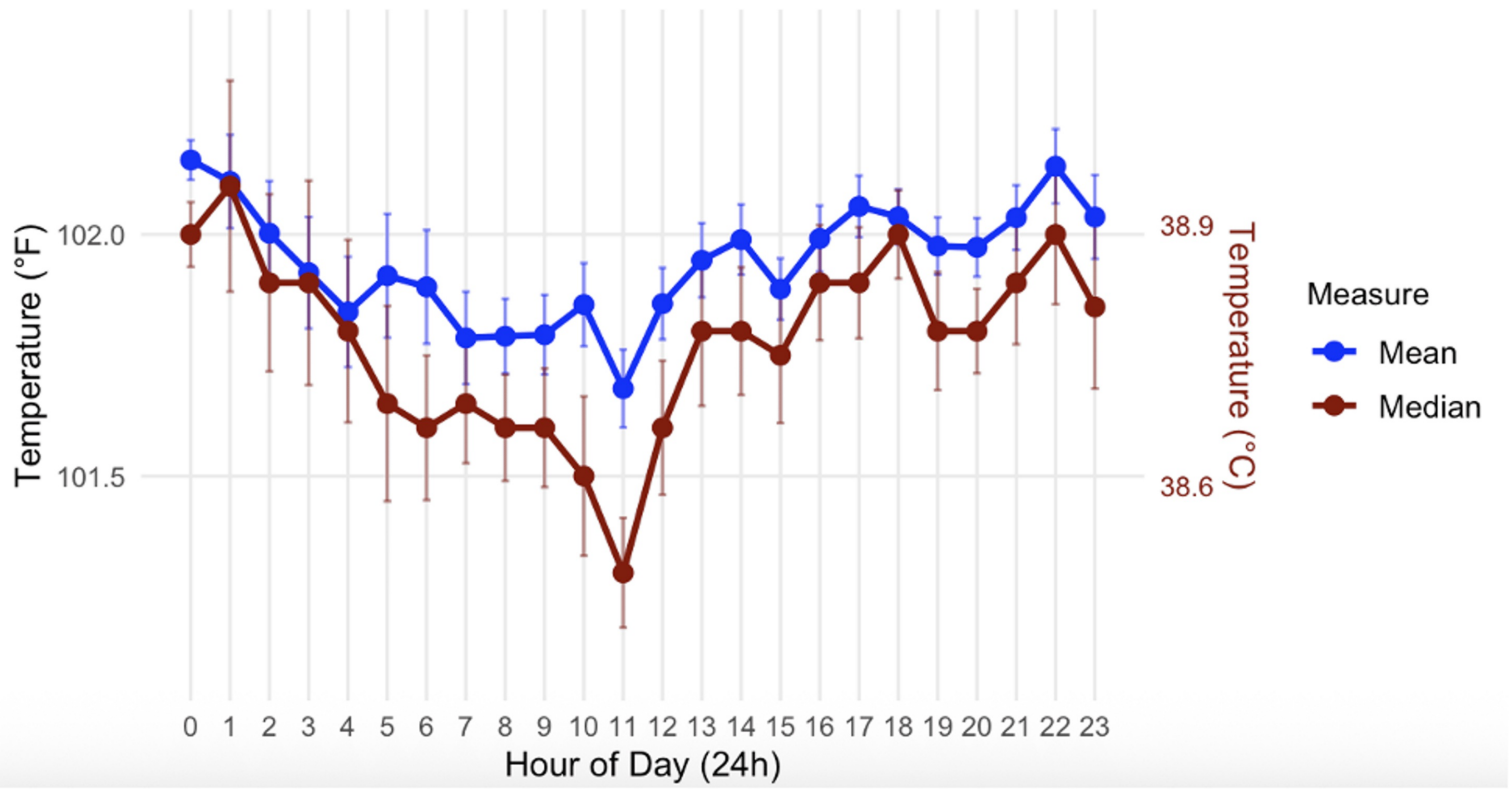
Temporal variation in hourly mean (± SE) and median BT over the 24 hours in 5,953 pyretic children aged six months to six years presenting to the PED. BT was highest in children presenting between 22:00 and 02:00 h and lowest between 07:00 and 11:00 h. The children likely followed typical sleep-wake routines, with sleep commencing for six-month-olds to one-year-olds at 18:30-19:30 h, for two- to three-year-olds at 19:00-20:00 h, and for six-year-olds at 19:00-21:00 h, alternating with daytime wakefulness likely commencing in all groups at 06:00-07:00 h, and diurnal naps lasting up to three hours in two-year-olds and ~1-1.5 hours in four-year-olds. The amplitude (peak-to-trough variation) over 24 hours is greater in the median than mean values. Clock time is shown on the x-axis in 24-hour format (6 = 6 a.m., 13 = 1 p.m., etc.), and BT is denoted on the y-axis in °F (left) and °C (right). PED: pediatric emergency department; BT: body temperature

Figure 1 illustrates the prominent sinusoid-like temporal variation in both the hourly mean (± SE) and median BT values of the entire cohort of pyretic children. Pyrexia, in terms of the hourly mean BT values, was least severe in children presenting to the PED between 07:00 and 11:00 h and most severe among those presenting between 22:00 and 02:00 h. Cosinor analysis of the hourly BT means shown in Figure 1 substantiates statistically significant 24-hour sinusoidal-like variation (p < 0.0001), with model-estimated MESOR (24-hour time series mean) of 101.9°F (~38.83°C), amplitude (total peak-to-trough difference) of 0.28°F (~0.16°C), and acrophase (clock time when children presented to the PED with the highest pyrexia) of ~21:15 h. A similar temporal pattern was observed for the hourly median BT values; however, the extent of variation between the highest values late during the night and the lowest values early during the day was greater. Cosinor analysis of the hourly median BT data again substantiated statistically significant 24-hour variation (p < 0.0001), with model-estimated MESOR of 101.8°F (~38.78°C), amplitude of ~0.42°F (~0.23°C), and acrophase of ~21:45 h.

Annual (seasonal) variation in BT

**Figure 2:**
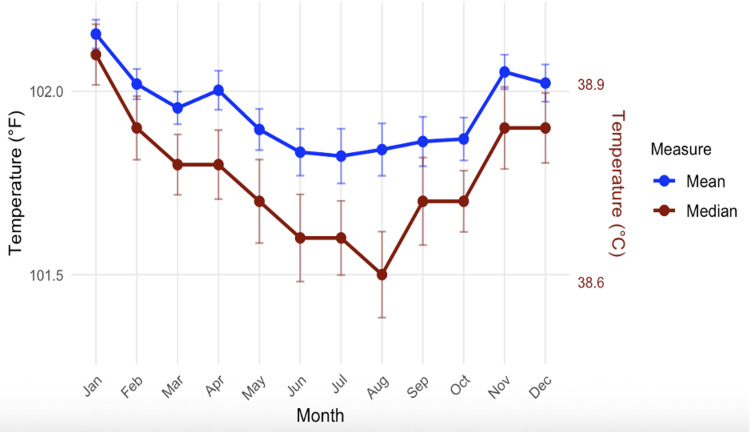
Temporal variation in the monthly mean (± SE) and median BT values of pyretic children aged six months to six years presenting to the PED. The BT means and medians of the pyretic children were highest in children presenting during the winter months of November, December, and January and lowest during the summer months of June, July, and August. The amplitude (total peak-to-trough difference) of the annual variation was greater for median than for mean values. Months of the year are denoted along the x-axis, and BT in °F (left) and °C (right) on the y-axis. PED: pediatric emergency department; BT: body temperature

Figure 2 displays the temporal pattern in the monthly mean (± SE) and median BT values of the cohort of pyretic children presenting to the PED at different times of the year. The monthly mean BT data exhibited sinusoidal-like temporal variation, with the highest values observed among children presenting to the PED during the winter months of November, December, and January and the lowest ones observed among children presenting to it during the summer months of June, July, and August. Cosinor analysis of the mean monthly values substantiated statistically significant annual variation (p = 0.0005), with model-estimated MESOR of 101.9°F (~38.83°C), amplitude of ~0.26°F (~0.14°C), and acrophase in January. The monthly median BT values exhibited a similar temporal patterning, but with greater amplitude of variation. Cosinor analysis of the median monthly values corroborated statistically significant annual variation (p = 0.0001), with model-estimated MESOR of 101.8°F (~38.78°C), amplitude of ~0.42°F (~0.23°C), and acrophase in January.

## Discussion

According to the time-of-day presentations to the PED, the severity of pyrexia in children six months to six years of age displayed sinusoidal-like 24 h patterning, which supports the hypothesis that the BTCR likely persists in the febrile state. However, the form of the 24-hour pattern differed somewhat from that observed in like-aged afebrile children: peak BT occurred in the late evening rather than late afternoon/early evening, and the amplitude of BT variation was less than ~1°C [15].

Results of the cross-sectional investigations - the current one by us [33,34] and by Harding et al. [37] - support the hypothesis that the endogenous BTCR likely persists in the febrile state, both in adults and children. Harding et al. [37] analyzed over 93,000 triage BT measurements from Beth Israel Deaconess Medical Center and more than 264,500 measurements from the 2002-2010 National Hospital Ambulatory Medical Care Survey (NHAMCS). The number of persons attending the Beth Israel Deaconess Medical Center expressing pyrexia, a triage BT ≥38.0°C, between 19:00 and 20:59 h was 2.5-fold greater than that between 07:00 and 08:59 h. Comparable findings were observed when the threshold of fever was redefined as ≥39.0°C or ≥40.0°C; the number of persons who exhibited fever at these levels was, respectively, 2.4- and 3.6-fold greater among those presenting between 19:00-20:59 h and 07:00-08:59 h. Analysis of the NHAMCS temperature data yielded almost identical findings. Evaluation of the data from our pediatric cohort revealed a 24-hour pattern in the severity of pyrexia comparable to that reported in adults by Harding et al. [37]. Moreover, the number of children displaying a BT greater than 102°F (~38.89°C), 103°F (~39.44°C), and 104°F (40.0°C) was 2.4-, 3.1-, and 2.6-fold greater, respectively, among those presenting to the PED between 21:00 and 01:00 h than between 07:00 and 11:00 h. Multiple studies of both children and adults, presumably adhering to the schedule of daytime wakefulness and nighttime sleep, demonstrate that the BTCR likely persists during febrile illness, with peak fever severity occurring in the late afternoon to evening period. In children with malaria, Lell et al. [19] reported substantially higher rates of pyrexia in the early evening compared with morning hours. Similar findings have been observed in adults across diverse medical conditions and clinical settings, including hospitalized cancer patients, surgical patients, and emergency care populations, where febrile episodes were several-fold more likely to be detected in the evening than in the morning [20,27,29-31]. Collectively, these studies consistently show that fever detection in normally day-active children and adults is lowest during early morning and daytime hours and highest between approximately 16:00 and 22:00 h, supporting recommendations to assess for pyrexia near the circadian peak of BT. Although rare exceptions have been reported [28], the preponderance of evidence [19-32] supports the persistence of circadian modulation of fever severity across age groups and clinical contexts.

We additionally demonstrated annual periodicity in the severity of pyrexia among children aged six months to six years presenting to the PED during different months of the year. Fever severity was highest among children evaluated in November, December, and January and lowest among those seen in June, July, and August. The annual pattern of BT in afebrile patients reported by Harding et al. [9], based on their analysis of a large database of emergency medical departments in the United States, differs remarkably from our findings in young febrile children. They reported average BT was lower, not higher, by 0.2°C during winter than summer, due to the influence of seasonal variation in the external thermal environment rather than the expression of an endogenous biological rhythm [9]. The existence of an innate basis for the annual patterning of the severity of fever in individual patients is uncertain because of the inability to conduct longitudinal investigations of sufficient duration that would require the unlikely persistence of pyrexia for a year or more. Most likely, the origin of the seasonal variation of pyrexia reported in children of our cohort derives from the annual variation in the manifestation and intensity of infectious childhood diseases. The number of children who presented to the PED having a triage BT >102°F (~38.89°C), 103°F (~39.44°C), and 104°F (40.0°C) was 3-, 3.7-, and 2.5-fold greater, respectively, during the winter months of November through February than during the summer months of June through August.

The results of our cross-sectional analysis of BT in young children closely align with those reported in the cross-sectional investigation of Harding et al. [37] and several longitudinal investigations that measured BT either continuously or frequently over one or more 24-hour periods [19-32]. Collectively, the results of studies conducted by us and others support the hypothesis that the BTCR persists in young pyretic children. Severity of fever was highest in children presenting to the PED between 22:00 and 02:00 h and lowest in those presenting between 07:00 and 11:00 h.

Limitations

Although this investigation exhibited strengths - i.e., a sufficiently large and high-quality database - the findings may have been compromised by certain weaknesses and uncontrollable factors.

First, the time-of-day pattern of presentations by children of different severities of pyrexia might not be entirely representative because of data loss. Parents and caretakers who had a highly reactive response to the initial signs of illness and fever in their child may have immediately sought care from office pediatricians during the daytime, while less reactive ones, who adopted a “wait-and-see” approach that resulted in children progressively developing more severe pyrexia during the day, sought care from PED clinicians in the evening.

Second, assessment of BT was recorded in the medical records in terms of external clock time rather than a biomarker of internal circadian time (e.g., hours before or after the main sleep span of each child). Clock time does not denote circadian time, which is deterministic of the features of the BTCR, particularly its acrophase. We presumed the pyretic children of our cohort likely adhered to the prescribed routine of nighttime sleep - between 18:30 and 19:30 h for six-month-olds to one-year-olds, 19:00-20:00 h for two- to three-year-olds, and 19:00-21:00 h for six-year-olds - alternating with daytime waking commencing between 06:00 and 07:00 h that served as the primary synchronizer of their BT and other circadian rhythms. However, this synchronizer schedule might have varied from the one presumed and additionally between the different children of our cohort. This might explain why the timing of the acrophase (i.e., ~21:15 h for the hourly mean values and 21:45 h for the hourly median values) was somewhat later than expected. Furthermore, discrepancies between children in the phasing of the BTCR might have masked detection of its true amplitude, perhaps explaining why the amplitude of BTCR was lower than expected, i.e., 0.28°F (~0.16°C) for the hourly mean values and ~0.42°F (~0.23°C) for the hourly median values.

Third, our observational investigation was cross-sectional in design. It relied on single-time-of-day BT measurements obtained from a large number of children of diverse race and socioeconomic status presenting with fever of various etiologies, with influenza and respiratory infections being the most prominent ones.

Fourth, the severity of the assessed BT of an unknown number of children might have been lessened by the provision, perhaps in a time-of-day-dependent manner, of antipyretics by parents/caretakers in the hours prior to the PED encounter.

Finally, the threshold used to diagnose pyrexia - triage, temporal, or oral temperature ≥100.4°F (38°C) - was fixed and unadjusted for the inherent, predictable-in-time circadian variation in BT characteristic of afebrile children aged six months and older. Because early morning BT is, on average, ~1°C lower than in the late afternoon/early evening, it is conceivable that an unknown number of children presenting to the PED in the morning - whose BT was abnormally elevated for that time of day but still below the established threshold temperature for fever - may have been classified as afebrile even though, in fact, they were biologically febrile.

## Conclusions

Our results, together with those of prior studies, infer that the severity of pyrexia assessed by a single time-of-day PED triage BT measurement is unlikely to be indicative of the severity of pyrexia at other times of the day and night. Future investigations are warranted to determine whether the BTCR becomes disrupted or dissipates in patients with certain pathologies and the extent, if any, to which such disruption foretells the need for special medical intervention and/or different patient outcomes. Finally, understanding the BTCR of pyrexia can provide valuable clinical insights for parental education and counseling, as well as more effective resource allocation to optimize staffing, improve triage strategies, and enhance patient care.
